# Multilayer Dye Aggregation at Dye/TiO_2_ Interface via π…π Stacking and Hydrogen Bond and Its Impact on Solar Cell Performance: A DFT Analysis

**DOI:** 10.1038/srep35893

**Published:** 2016-10-21

**Authors:** Lei Zhang, Xiaogang Liu, Weifeng Rao, Jingfa Li

**Affiliations:** 1 Department of Applied Physics, School of Physics and Optoelectronic Engineering, Nanjing University of Information Science and Technology, No. 219 Ning Liu Road, Nanjing 210044, China; 2 Singapore-MIT Alliance for Research and Technology (SMART) Centre, 1 Create Way, 138602, Singapore; 3 Department of Materials Physics, School of Physics and Optoelectronic Engineering, Nanjing University of Information Science and Technology, Nanjing 210044, China

## Abstract

Multilayer dye aggregation at the dye/TiO_2_ interface of dye-sensitized solar cells is probed via first principles calculations, using *p*-methyl red azo dye as an example. Our calculations suggest that the multilayer dye aggregates at the TiO_2_ surface can be stabilized by π…π stacking and hydrogen bond interactions. Compared with previous two-dimensional monolayer dye/TiO_2_ model, the multilayer dye aggregation model proposed in this study constructs a three-dimensional multilayer dye/TiO_2_ interfacial structure, and provides a better agreement between experimental and computational results in dye coverage and dye adsorption energy. In particular, a dimer forms by π…π stacking interactions between two neighboring azo molecules, while one of them chemisorbs on the TiO_2_ surface; a trimer may form by introducing one additional azo molecule on the dimer through a hydrogen bond between two carboxylic acid groups. Different forms of multilayer dye aggregates, either stabilized by π…π stacking or hydrogen bond, exhibit varied optical absorption spectra and electronic properties. Such variations could have a critical impact on the performance of dye sensitized solar cells.

Dye sensitized solar cells (DSSCs) utilize molecular dye absorbers to convert photo energy into electrical power^1^,^2^,^3^,^4^. The dye/TiO_2_ interface in the working electrode of a DSSC is responsible for light absorption and charge separation in a DSSC^5^,^6^. Various researches have been carried out to understand the interfacial dye/TiO_2_ structures at nanoscale toward formulating structure-property relationship of the photoelectrode and optimizing DSSC performance^7^,^8^.

Substantial first principles calculations have been performed in order to establish an accurate model to describe the nanoscale structures of dye/TiO_2_ interface^8^,^9^,^10^,^11^. Monomeric dye adsorption on TiO_2_ surface has been extensively evaluated for various dyes to obtain adsorption geometry and dye/TiO_2_ binding mode^12^,^13^,^14^. Yet, the monomeric dye adsorption model is often inadequate to describe the dye/TiO_2_ interface; this is due to the fact that many dye molecules are readily adsorbed on the TiO_2_ surface and form closed packed dye aggregates via intermolecular interactions^15^,^16^. It has been identified that molecular dye aggregates are responsible for novel optoelectronic properties for functional molecular dyes in DSSCs and related devices, and numerous researches have been performed to manipulate molecular aggregates and fine-tune their materials properties^17^,^18^,^19^,^20^,^21^,^22^. To this end, dimeric dye aggregates at different anchoring positions on the TiO_2_ surface have been analyzed by Filippo *et al.*^23^, offering nanoscopic insights on experimental phenomena that is inconsistent with the monomeric adsorption model. To more closely reassemble experimental scenarios, higher degrees of dye aggregates, such as trimer, tetramer and pentamer, have been constructed via first principles calculations^24^. These aggregation models qualitatively explain the “H-aggregation” phenomena of dyes at the dye/TiO_2_ interface, where blue-shifted light absorption spectra are observed. However, the existing aggregation models are limited in the lateral direction relative to the TiO_2_ surface, while dye aggregate structures built outward from the TiO_2_ surface are neglected. In other words, a monolayer model is assumed. The monolayer model sets an intrinsic limitation on the amount of dye adsorption on TiO_2_ surface, leadings to theoretical predications that are two orders of magnitude smaller than the experimental results^8^,^24^. An improved multilayer model to describe the interfacial dye aggregates is thus required to bridge the gap between experimental and computational results.

Azo dyes are frequently used in textile and coating industries due to its stability, vivid color, high molar extinction, and low cost^25^,^26^,^27^. Recently, it has been successfully incorporated into DSSCs and convert light into electricity^28^,^29^. The aggregation of *p*-methyl red azo dyes has been evaluated in previous research in the lateral direction relative to the TiO_2_ surface^24^, and the study on the *p*-methyl red azo dyes serves as a good platform to further analyze their intermolecular interactions and thereby construct the multilayer aggregation model.

In this manuscript, *p*-methyl red [4-[2-[4-(dimethylamino)phenyl]diazenyl]-benzoic acid] is used to probe the dye aggregation on TiO_2_ surface (Fig. 1). The nanoscale structures of different dye aggregates stabilized via intermolecular forces will be presented, followed by a detailed discussion on their electronic and optical properties that impact solar cell performance.

## Results and Discussion

### Structure of dye adsorbates on a (101) TiO_2_ surface

**Figure 2:**
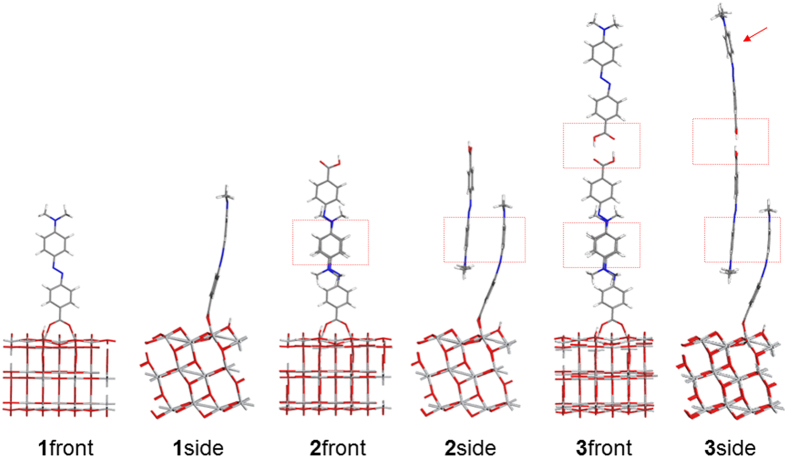
Optimized geometries of monomer **1**, dimer **2** and trimer **3** of methyl red molecules. Both front view and side view are depicted for clarify. The π…π stacking and hydrogen bond interactions are highlighted in red rectangles. The red arrow points to a potential adsorption site for further aggregate expansion via π…π interaction.

In the optimized structure of the azo dye/TiO_2_ (Fig. 2), the molecular axis of the adsorbates lies perpendicular to the TiO_2_ surface. Two molecules in dimer **2** are stabilized by π…π interactions. The top azo molecule in dimer **2** has it carboxylic acid group facing outward away from the TiO_2_ surface. The separations between two oxygen atoms in the carboxylic acid group and TiO_2_ surface are similar among **1**–**3** (2.08 Å, 2.07 Å and 2.05 Å for **1**–**3**, respectively), consistent with previously reported values in the literature^24^. The bond lengths of the azo group (-N=N- bond), an indicator of intramolecular charge transfer^30^, are also similar (1.27–1.28 Å) in **1**–**3**. The π…π distance between the two azo molecules are 4.1 Å for both aggregates **2** and **3**, while the H…O distance of the hydrogen bond is 1.53 Å for **3**. Interestingly, the π…π stacking mode and the hydrogen bond interactions between neighboring carboxylic acid groups have been observed in the crystal structures of azo dyes determined from single crystal X-ray diffraction experiment^29^. The monomeric form **1** demonstrates more perpendicular adsorption geometry compared with **2** and **3**. The angle between its molecule axis and TiO_2_ surface is 3°–4° larger in **1** than that in **2** and **3**. The molecule chemisorbed onto TiO_2_ surface is more distorted in the case of **2** and **3**, due to steric effects introduced by additional azo molecules.

### Dye adsorption energies and the amount of dye loading on TiO_2_ surface

The adsorption energy of the three aggregates follows the ranking: **1** (0.60 eV) >**3** (0.56 eV) >**2** (0.43 eV; Table 1). This is because **1** possesses chemisorption which is more stable in nature than physisorption. **2** is the most unstable system because the intermolecular π…π interaction is based on relatively weak van der waals forces. Molecules in **3** are connected by hydrogen bond which has interaction strength between chemical bonding and van der waals forces.

In terms of dye coverage, **3** has the highest degree of aggregation (0.43 nmol/cm^2^) among **1**–**3** because it involves three molecules in the unit cell. In contrast, **1** demonstrates the lowest dye coverage (0.14 nmol/cm^2^). The experimental dye coverage is 147 nmol/cm^2^ for *p*-methyl red on TiO_2_ nanoparticles in a DSSC photoelectrode^8^. Although a large discrepancy exists between experimental and calculated values, it is of note that **3** provides adsorption sites for further vertical aggregate expansion (Fig. 2). The outermost molecule in **3** with respect to TiO_2_ surface can interact with a forth molecule via π…π interactions (indicated by a red arrow in Fig. 2); so on and so forth. In this way, the π…π stacking and hydrogen bond interactions offers a route to construct periodic structures in the *c*-axis, leading to much higher dye loading. As a result, the multilayer aggregation model proposed in this manuscript agrees better with the experimental results^8^. In contrast, the monolayer model in the literature produces a maximum dye coverage of 0.42 nmol/cm^2^ for *p*-methyl red on TiO_2_ 2D slab^8^, three orders of magnitude smaller than experimental values.

We also noticed that after rinsing dye/TiO_2_ photoelectrodes during DSSC fabrications (using washing solvent, such as ethanol, to remove weakly adsorbed dye molecules during the sensitization process), the washing solvent displays vivid color due to dye dissolution; this phenomenon indicates that abundant azo dyes are weakly adsorbed on the TiO_2_ surface such that they can be easily removed from TiO_2_ substrate. Previous aggregation models, that involve only stable chemisorption, are not sufficient to explain these experimental observations. In contrast, the multilayer aggregation model which incorporates physisorption (such as π…π interaction and hydrogen bond) provides a plausible explanation to the large amount of weakly adsorbed azo dyes on TiO_2_ substrates.

### Optical Properties

**Figure 3:**
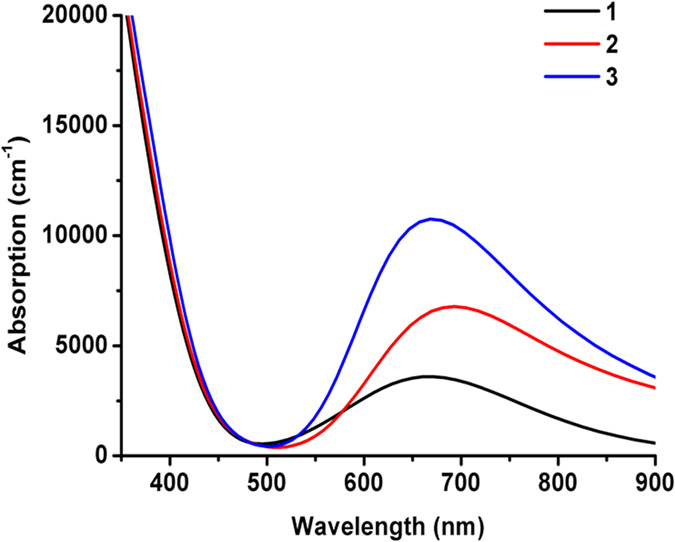
The calculated UV/vis absorption spectra of **1**–**3** with *p*-methyl red dye adsorbates on TiO_2_ substrate predicted by PBE functional.

The calculated UV/vis absorption spectra of **1**–**3** exhibit two major bands: one in the UV region near 400 nm and the other in the visible region from 500 nm to 900 nm (Fig. 3). The intense band in the UV region corresponds to the light absorption in TiO_2_ substrate, while the visible band corresponds to the absorption of the dye adsorbates. Dimer **2** has the most red-shifted visible band (with peak wavelength at 693 nm). This peak wavelength is red shifted by 22 nm compared with that of monomer **1**, demonstrating that the π…π stacking interaction is effective in tuning optical properties of DSSC (a red shifted UV/vis absorption spectra is desirable for DSSC device performance for a close match with solar spectrum). Trimer **3** stabilized by hydrogen bond between molecules exhibits the most blue-shifted light absorption spectra among **1–3**, indicating that hydrogen bond between neighboring carboxylic acid anchoring groups in azo dyes is detrimental for improving the light absorption of a DSSC device. The experimental light absorption spectra of the *p*-methyl red/TiO_2_ film exhibit severe H-aggregation and its peak wavelength approaches the UV region at 400 nm^8^. As a result, the blue-shifted absorption spectrum of **3** implies that the aggregate **3** represents a possible interfacial structure. Further aggregation expansion, which is computationally expensive and unreachable at this stage, is expected to further narrow the gap between experimental and theoretical results. Note that the aggregate **3** does not conform to the typical H-aggregate model, which usually exhibit a head-to-head and tail-to-tail conformation. Instead, the direct “face-to-face” interactions via the hydrogen bonds in aggregate **3** essentially lead to partial dipole-dipole cancellation, because both carboxylic acid groups carry partial negative charge as a result of intramolecular charge transfer in azo dyes^29^,^31^. The net result of such interactions affords a blue-shift^30^,^32^ in the light absorption spectra of aggregate **3**.

We also notice that the calculated UV/vis spectra of **1–3** (~600 nm) demonstrate a large red shift against experimental data (~400 nm), which has also been experienced in the simulation based on the monolayer model^8^. This is not surprising, because the pure functional PBE (used in our study) neglects Hartree-Fock like exact exchange and tends to severely underestimate the band gap and the excitation energies of charge-transfer dyes (such as azo dyes)^33^,^34^,^35^. Nevertheless, the relative spectral difference among **1**–**3** is more reliable and provides a strong support to our multi-layer aggregation model.

### Band Structure and Density of States

**Figure 4:**
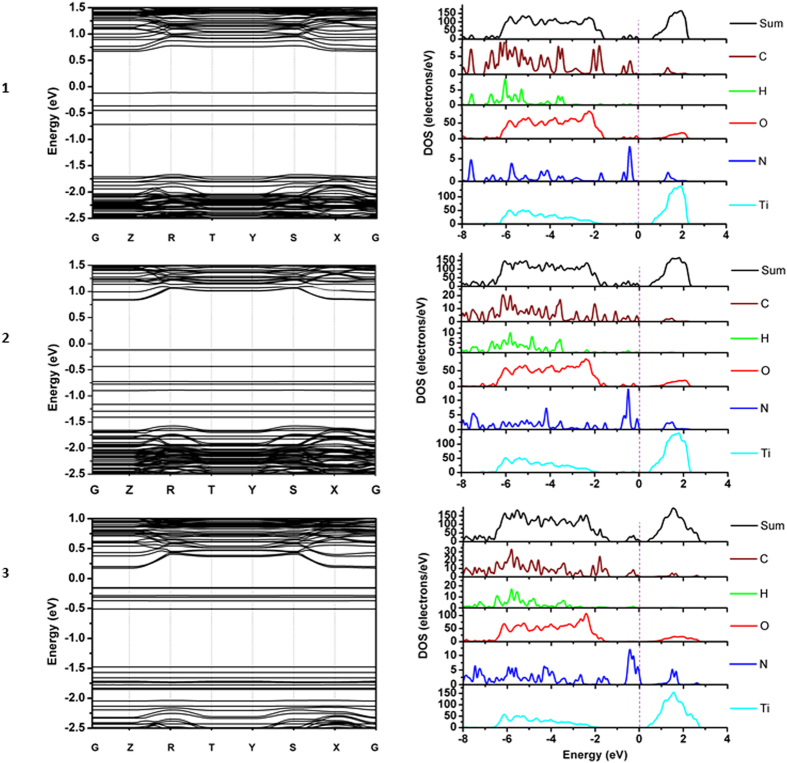
Band Structures, and projected density of states (PDOS) of C (carbon), H (hydrogen), O (oxygen), N (nitrogen) and Ti (titanium). The vertical dotted lines correspond to the Fermi Energy, which is set to 0 eV.

The band structures of **1**–**3** exhibit a series of straight band lines below Fermi level (Fig. 4). These straight band lines are discrete and are contributed by organic molecules^36^. Nevertheless, these band lines are only defect states and do not affect the electronic band gap of the material. **2** has dense band lines near valence bands contributed by organic dyes. The band lines in **2** are denser than those in **1**, due to more atoms available in **2**. As a result, the optical absorption spectra, which are related to the difference between conduction band and valence band^24^, are expected to demonstrate a red-shift in **2** with respect to that in **1**. In the case of **3**, most bands contributed by the chromophore are localized in the deep region between −1.5 and −2.0 eV, which are further away from the conduction band. This could explain the blue shifted absorption spectrum of **3** compared with that of **2**. The band lines with large curvature in the upper and lower regions of the band structure plots are contributed by periodic TiO_2_ 2D slab.

In the PDOS spectra (Fig. 4), the HOMO (highest occupied molecular orbital) is contributed by carbon, oxygen and nitrogen atoms in the organic molecules. The deep valence bands between −6 eV and −2 eV are mainly contributed by TiO_2_ oxygen atoms. The conduction band is mainly contributed by Ti atoms in the TiO_2_ substrate. These features are common in a dye/TiO_2_ system.

### Orbital Distributions

**Figure 5:**
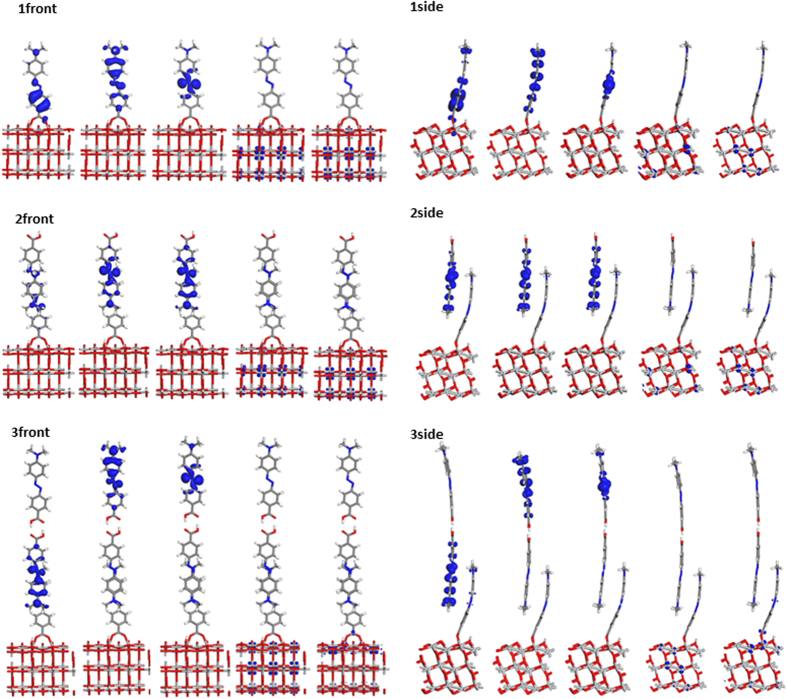
Orbital distributions of the dye/TiO_2_ systems. Five orbitals are presented from left to right of each structure: HOMO-2, HOMO-1, HOMO, LUMO and LUMO+1.

The orbital distributions for molecular orbitals HOMO-2, HOMO-1, HOMO, LUMO, and LUMO+1 are depicted to provide an intuitive visualization on the electronic properties of the dye/TiO_2_ system (Fig. 5). Electron distributions in the unoccupied orbitals are almost the same in **1**–**3**: the LUMO and the LUMO+1 are distributed in the TiO_2_ region, displaying Ti 3d characters. The occupied molecular orbitals vary among the three structures: for **1**, HOMO is localized in the azo group of the molecule; HOMO-1 is delocalized across the entire azo molecule; HOMO-2 is in the aromatic ring next to the TiO_2_ surface. In the case of **2**, the HOMO-2, HOMO-1 and HOMO are localized in the upper molecule interacting with the rest of the system via π…π stacking forces. As a result, this additional molecule in **2** plays an important role in the electronic and optical properties when azo dyes are incorporated in a DSSC device. In the case of **3**, HOMO and HOMO-1 are distributed in the uppermost molecule interacting with the rest system via hydrogen bond, while HOMO-2 resides in the middle molecule. For all the three structures, the outermost molecule is the most active in electronic properties due to HOMO localization in this region, thus having an important impact on the electronic and optical properties of the dye/TiO_2_ system.

## Conclusions

In this study, we propose a multilayer dye aggregation model to describe the aggregation phenomena in the dye/TiO_2_ interface. We show that multilayer aggregates may form along the *c*-axis of the dye/TiO_2_ unit cell via intermolecular interactions, such as π…π stacking and hydrogen bond interactions. Our calculations suggest that further aggregation growth may take place in the *c*-axis, forming a large 3D dye/TiO_2_ interfacial structure. This multilayer dye aggregation model could thus bridge the gap between theoretical and experimental dye loading on TiO_2_ surface, in contrast to the monolayer dye adsorption model. Moreover, the weak adsorptions of azo dyes on TiO_2_ substrate, reported in previous experiments, can be rationalized in the new multilayer model in terms of weak physisorption (in contrast to strong chemisorption in the monolayer model). We also investigated the spectral and electronic properties of the multilayer molecular aggregates as well as their impacts on the DSSC device performance. We show that the π…π stacking induce a red shift, while hydrogen bond leads to a blue shift of the light absorption spectra in the dye/TiO_2_ system. It is expected that this multilayer model will pave a new way for a deeper understanding of large molecular aggregation systems in DSSC and other relevant applications.

## Methods

A unit cell of (TiO_2_)_36_ exposing anatase 101 surface is constructed to analyze the multilayer dye aggregation. A very long vacuum layer and *c*-axis (69.4 Å) of the unit cell is built in order to accommodate the multiple dye molecules in the *c*-axis extending outward from the TiO_2_ surface. A bidentate bridging anchoring mode for the carboxylic acid anchoring group on anatase 101, suggested by previous research^37^,^38^, is used in this project. Three types of aggregates **1**–**3** are investigated in this study, where **1** represents the monomeric adsorption form; **2** is a dimer joined via π…π stacking effects; **3** is a trimer where a third molecule is placed on top of dimer **2** via hydrogen bond. Upon adsorption, the vacuum layer thickness values are ca. 44 Å for **1**, 39 Å for **2**, and 23 Å for **3**, which are sufficient to minimize interactions between neighbouring layers^39^,^40^,^41^,^42^,^43^,^44^. A 1 × 3 × 1 supercell was constructed to understand the multilayer aggregation, corresponding to a surface area of 10.2 × 11.3 Å^2^. The three interfacial structures **1**, **2** and **3** are geometrically optimized in VASP^45^, using PBE functional (energy cutoff: 500 eV; ionic displacement convergence criterion: 0.02 eV/Å). During geometric optimization, oxygen atoms at the bottom of the unit cell are frozen while all other atoms are freely relaxed to their equilibrium positions. The band structures and density of states of **1**–**3** are calculated via an 8 × 8 × 1 monkhorst-pack grid. The optical absorption spectra are obtained from dielectric constant in Castep^46^, using 430 eV as the energy cutoff. Graeme D2 dispersion correction^47^ is included in all the calculations. The dye adsorption energy on TiO_2_ surface is calculated using:


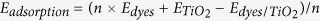


The first term in the bracket corresponds to the total energy of molecular dyes; the second is the total energy of bare TiO_2_ substrate, and the third term is the total energy of the dye/TiO_2_ system; and *n* is the number of dye adsorbates.

## Additional Information

**How to cite this article**: Zhang, L. *et al.* Multilayer Dye Aggregation at Dye/TiO_2_ Interface via π…π Stacking and Hydrogen Bond and Its Impact on Solar Cell Performance: A DFT Analysis. *Sci. Rep.* **6**, 35893; doi: 10.1038/srep35893 (2016).

## Figures and Tables

**Figure 1:**
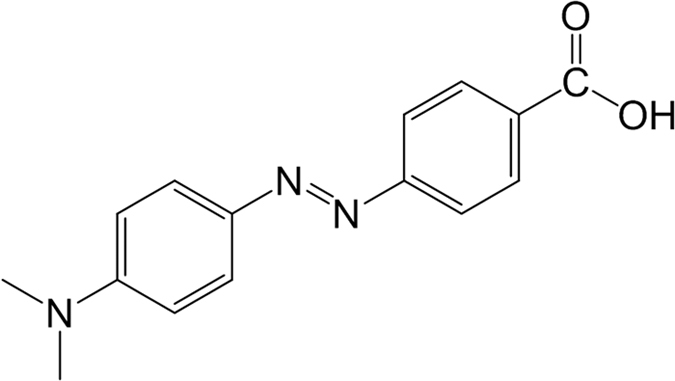
The molecular structure of 4-[2-[4-(dimethylamino)phenyl]diazenyl]-benzoic acid (*p*-methyl red).

**Table 1:** Estimated adsorption energies and dye coverage levels on the (101) TiO_2_ surface for different aggregates.

	1	2	3
Adsorption Energy (eV)	0.60	0.43	0.56
Dye Coverage (nmol/cm^2^)	0.14	0.29	0.43
